# Clinicopathological characteristics of thyroid cancer in the federal state of Salzburg

**DOI:** 10.1007/s00508-017-1207-x

**Published:** 2017-05-10

**Authors:** Gundula Rendl, Margarida Rodrigues, Gregor Schweighofer-Zwink, Josef Hutter, Anton Hittmair, Barbara Zellinger, Cornelia Hauser-Kronberger, Christian Pirich

**Affiliations:** 1 Department of Nuclear Medicine and Endocrinology, Paracelsus Medical University Salzburg, Müllner Hauptstrasse 48, 5020 Salzburg, Austria; 2 Department of Nuclear Medicine, Schwarzach Hospital, Schwarzach, Austria; 3 Department of Pathology, Schwarzach Hospital, Schwarzach, Austria; 4 Department of Pathology, Paracelsus Medical University Salzburg, Salzburg, Austria

**Keywords:** Thyroid cancer, Endocrine system, Diagnosis, Gene mutation, Fine-needle aspiration biopsy

## Abstract

**Objective:**

The aim of our investigation was to evaluate the clinicopathological characteristics and mutation patterns in newly diagnosed cases of thyroid cancer in the federal state of Salzburg, Austria, in the year 2013.

**Methods:**

The medical records of all patients newly diagnosed with thyroid cancer in 2013 in the federal state of Salzburg were retrospectively reviewed. The clinicopathological characteristics and mutations of thyroid cancers were analyzed.

**Results:**

63 patients (mean age: 51.0 years, range: 21–81 years; female 75%, male 25%) were identified. 53 patients had papillary (12 follicular variant), 4 patients follicular (1 oxyphilic variant), 3 patients medullary, and 3 patients anaplastic thyroid cancer. T1 tumors were found in 34 patients (pT1a, 20 patients; pT1b, 14 patients), T2 tumors in 10 patients, T3 tumors in 16 patients, and T4 tumors in 3 patients. Lymph node involvement was seen in 15 patients and metastatic disease in 1 patient. Mutations of BRAF (B-type Raf kinase) were detected in 23 and mutation of NRAS (Neuroblastoma RAS Viral Oncogene Homolog) in 2 papillary thyroid cancers. No concomitant mutations of BRAF and NRAS were found.

**Conclusion:**

Females accounted for 75% of the patients with newly diagnosed thyroid cancer and the incidence peaked at a younger age than in males. Papillary thyroid cancer was the most frequent tumor type, accounting for 84% of the cases. A high frequency of T1 tumors and cancers with no lymph node involvement was found. Males had a higher proportion of large tumors and more aggressive forms of thyroid cancer than females. Mutations (mostly of BRAF) were found in 47% of the cases. Neither mutations of KRAS (Kirsten rat sarcoma viral oncogene homologue) nor concomitant mutations of BRAF and NRAS were found.

## Introduction

Thyroid cancer is the most frequent cancer among endocrine tumors. Its incidence has been increasing over the past several decades worldwide [1–3], showing considerable regional variation [4–9].

The increased incidence of thyroid cancer is often attributed to increased detection associated with better access to healthcare, diligent health screening with rising use of thyroid imaging, and optimized management strategies [1, 3, 8–10]. Small papillary thyroid cancers, including papillary microcarcinomas, have mainly contributed to the worldwide increased incidence of thyroid cancer observed over the past decades [1, 3, 5, 7]. However, the increasing number of large tumors and rates of thyroid cancer-related mortality in spite of earlier treatment, as well as the changes in thyroid cancer molecular profiles, also suggest a true increase in the number of cases. Hormonal influences, including increased iodine intake and chronic lymphocytic thyroiditis, and recently increased and thyroid-specific environmental carcinogens, including exposure to radiation and environmental pollutants such as nitrates, heavy metals, and other compounds largely used in the industrialized society [3, 11–13], could be responsible. Causal relationships require further experimental and epidemiological studies.

Thyroid cancer exists in several forms. Differentiated thyroid cancers include papillary and follicular thyroid cancer, and both arise from the thyroid follicular cells. Medullary thyroid cancer arises from the parafollicular cells. Anaplastic thyroid cancer is one of the most aggressive and rapidly fatal cancers. It can develop from differentiated thyroid cancer that de-differentiates over time, or can also arise de novo [14]. The prognosis and treatment of thyroid cancer depend on the tumor type and its stage at the time of diagnosis [13, 15]. Age, the completeness of resection, and extrathyroidal extension are prognostic indicators employed in many staging systems for differentiated thyroid cancer [13–15].

Fine-needle aspiration biopsy (FNAB) is the gold standard for the differential diagnosis of thyroid nodules, but has the limitation of inadequate sampling or indeterminate lesions. Molecular analysis of gene mutations was found to improve the diagnostic performance of FNAB [16].

The aim of the present investigation was twofold: first, to assess the clinicopathological characteristics of newly diagnosed thyroid cancers in the federal state of Salzburg, Austria, in the year 2013; and secondly, to analyze mutations in differentiated thyroid cancers. Furthermore, the differences in clinical routine leading to the first diagnosis of thyroid cancer in two endocrine centers were evaluated.

## Materials and methods

The medical records of all patients who were newly diagnosed with thyroid cancer (ICD10 code C73) during the year 2013 in the federal state of Salzburg were retrospectively reviewed. We compared the differences in clinical routine leading to thyroid gland surgery and diagnosis of thyroid cancer between two endocrine centers in the federal state of Salzburg.

The clinicopathological characteristics of all patients with thyroid cancer and gene mutation tests (performed for BRAF [B‑type Raf kinase], KRAS [V‑Ki-ras2 Kirsten rat sarcoma viral oncogene homolog], and NRAS [Neuroblastoma RAS Viral Oncogene Homolog]) in differentiated thyroid cancers were evaluated.

## Results

### Clinicopathological characteristics

A total of 63 patients with newly diagnosed thyroid cancer were identified retrospectively (cancer registry) in the federal state of Salzburg in 2013. 47 patients were female (75%) and 16 patients were male (25%). The mean age at diagnosis was 51.0 years (female, 48.3 years; male, 51.9 years; range: 21–81 years).

The differences in clinical routine in the two centers were that in center 1, patients with hypofunctional thyroid nodules or otherwise morphologically suspicious thyroid nodules underwent preoperative ultrasound-guided FNAB. In center 2, hypofunctional thyroid nodules or morphologically suspicious thyroid nodules only underwent preoperative FNAB, which was not ultrasound-guided, if the nodule was palpable.

In center 1, 33 patients (52%) were newly diagnosed with thyroid cancer in 2013. Out of these, 32 patients underwent thyroid surgery because of the following findings: 23 patients had suspicious thyroid nodules (20 patients underwent preoperative FNAB, which was positive for thyroid cancer in 17 patients and inconclusive in 3 patients), 8 patients showed growing nodules in multinodular goiter, and 1 patient had concomitant Graves’ and multinodular disease. The remaining 1 patient was not operated upon due to the diagnosis of anaplastic thyroid carcinoma.

In center 2, 30 patients (48%) were newly diagnosed with thyroid cancer in 2013. 28 of these patients underwent thyroid surgery due to the following findings: 25 patients had suspicious thyroid nodules (15 patients underwent preoperative FNAB, which was positive for thyroid cancer in 14 patients and inconclusive in 1 patient), 2 patients showed growing nodules in multinodular goiter, and 1 patient had recidivated Graves’ disease. The other 2 patients were not operated upon because of the diagnosis of anaplastic thyroid carcinoma.

Overall, 60 patients (95%) underwent thyroid surgery. Preoperative FNAB was applied in 38 patients (60%). It was positive for thyroid cancer in 34 patients (90%) and inconclusive in 4 patients (10%).

The histological types were papillary thyroid cancer in 53 patients (84%; follicular variant in 12 patients, 19%), follicular thyroid cancer in 4 patients (6%; oxyphilic variant in 1 patient, 2%), medullary thyroid cancer (all sporadic in genetic analysis for MEN IIa/IIb) in 3 patients (5%), and anaplastic thyroid cancer in 3 patients (5%). The tumors were unifocal in 50 patients (79%) and unilateral in 52 patients (83%).

**Fig. 1:**
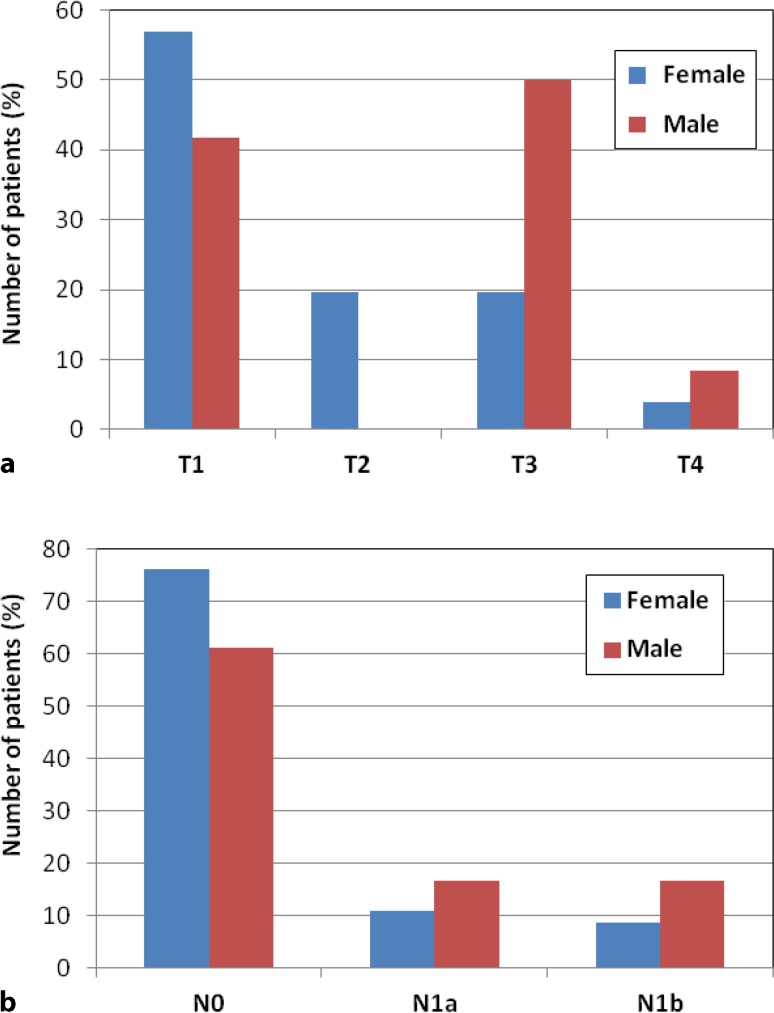
Distribution of **a** T‑ and **b** N‑stage across gender

T1 tumors were found in 34 patients (54%). Of these patients, 20 patients (32%) had a pT1a tumor and 14 patients (22%) had a pT1b tumor. T2 tumors were diagnosed in 10 patients (16%), T3 tumors in 16 patients (25%), and T4 tumors in 3 patients (5%). Lymph node involvement was seen in 15 patients (24%) and metastatic disease (pulmonary metastases) in 1 patient (2%) with papillary thyroid cancer (pT3 N0 M1). Table 1 shows the distribution of T‑stage and N‑stage across histological type, finding, focality, and center. Fig. 1 illustrates the distribution of T‑stage and N‑stage across gender. Female patients with all histological types peaked at a younger age than male patients, except patients with medullary thyroid cancer. Local and vascular invasion were found in 7 (11%) and 5 patients (8%), respectively.

**Table 1:** Distribution of T‑stage and N‑stage

	Histological type				Finding		Focality		Center	
	Papillary (*n* = 53)	Follicular (*n* = 4)	Medullary (*n* = 3)	Anaplastic (*n* = 3)	Suspicious (*n* = 48)	Incidentaloma (*n* = 15)	Unifocal (*n* = 50)	Multifocal (*n* = 13)	1 (*n* = 33)	2 (*n* = 30)
*T1a* (*n* = 20)	18	1	1	–	16	4	16	4	13	7
*T1b* (*n* = 14)	13	–	1	–	10	4	10	4	7	7
*T2* (*n* = 10)	8	2	–	–	8	2	8	2	4	6
*T3* (*n* = 16)	14	1	1	–	11	5	13	3	8	8
*T4* (*n* = 3)	–	–	–	3	3	–	3	–	1	2
*N0* (*n* = 45)	40	3	2	–	33	12	37	8	23	22
*N1a* (*n* = 9)	7	1	1	–	7	2	7	2	5	4
*N1b* (*n* = 6)	6	–	–	–	5	1	3	3	4	2
*Nx* (*n* = 3)	–	–	–	3	3	–	3	–	1	2

*n* number of cases

### Gene mutation analysis

Mutations of BRAF, KRAS, and NRAS were tested in tissue samples of 53 patients (39 papillary thyroid cancers, 10 with follicular variant; 3 follicular thyroid cancers, 1 oxyphilic variant).

BRAF mutations were detected in 23 cases with papillary thyroid cancer (43%). NRAS mutations (in codon 61) were found in 2 other cases with papillary thyroid cancer (4%; 1 case with follicular variant).

No case was found with overlapping mutation of BRAF and NRAS.

No mutations of BRAF and NRAS were found in 28 cases (53%; 16 patients with papillary thyroid cancer, 8 patients with follicular variant; 3 patients with follicular thyroid cancer, 1 patient with oxyphilic variant). Table 2 shows the distribution of BRAF and NRAS mutations across T‑stage and N‑stage.

**Table 2:** Distribution of BRAF and NRAS mutations across T‑stage and N‑stage

	BRAF		NRAS	
	BRAF mutations (*n* = 23)	No BRAF mutation (*n* = 30)	NRAS mutation (*n* = 2)	No NRAS mutation (*n* = 51)
*T1a* (*n* = 18)	11	7	–	18
*T1b* (*n* = 13)	6	7	1	12
*T2* (*n* = 9)	2	7	1	8
*T3* (*n* = 13)	4	9	–	13
*N0* (*n* = 39)	16	23	2	37
*N1a* (*n* = 8)	5	3	–	8
*N1b* (*n* = 6)	2	4	–	6

*n* number of cases, *BRAF* B-type Raf kinase, *NRAS* Neuroblastoma RAS Viral Oncogene Homolog

None of the 53 cases investigated showed a KRAS mutation.

No correlation between gene mutations and gender, age, or TNM classification of tumors was found.

## Discussion

In Austria, despite the introduction of iodine goiter prophylaxis in the early sixties, multinodular goiter remains endemic [17]. Both gender distribution and peak age at diagnosis of thyroid cancer in the federal state of Salzburg are similar to those reported in the literature [1, 3, 5, 8]. In our population the proportion of T1 tumors and cancers with no lymph node involvement was high (54 and 76%, respectively). These favorable pathological characteristics may be attributable to the wide use of imaging studies—including ultrasound, computed tomography, magnetic resonance imaging, and positron-emission tomography scans—that incidentally detect thyroid nodules, as also found by other investigators [9, 10]. Furthermore, FNAB (which was inconclusive in only 10% of this series of patients) makes an important contribution to decision making in the management of suspicious thyroid nodules and earlier diagnosis of thyroid cancer. Consistently, the proportion of T1 tumors found in center 1 (where all patients with hypofunctional thyroid nodules or otherwise morphologically suspicious thyroid nodules underwent preoperative ultrasound-guided FNAB) was higher than that seen in center 2. However, it is noteworthy that in our series, both the proportion of T3 and T4 tumors, and the numbers of cancers with extrathyroidal invasion were higher in male than in female patients. These pathological differences found between female and male patients suggest that other influences besides optimized diagnosis could be responsible for the increased incidence of thyroid cancer.

Local lymph node invasion was found in 24% of patients of our series. These results support the further use of single-photon-emission computed tomography (SPECT)/computed tomography (CT) in the follow-up of thyroid cancer patients.

In our study population, the proportion of patients with papillary thyroid cancers, in particular with T1 tumors, was high. This finding is consistent with the reports of a prominent increase in small, papillary thyroid cancers in several regions [1, 3, 5, 7, 18]. This might be associated with a high iodine intake, which is reported to be related to the development of papillary thyroid cancers [1, 19, 20]. Neuhold et al. [17] suggested that the levels of iodine intake play only a minor role in the early phase of carcinogenesis of papillary thyroid cancer, but may be of some importance during the progression of latent to clinically evident papillary thyroid cancer. However, the association between thyroid cancer and iodine intake remains unclear.

Thyroid cancers have been found to harbor multiple gene mutations or rearrangements [16, 21–25]. Cantara et al. [16] reported that the presence of mutations at cytology was associated with cancer 91% of times and follicular adenoma 9% of times. BRAF mutations were always associated with cancer [16], whereas RAS mutations were mainly associated with cancer (74–88%) [16, 21], but also with follicular adenoma (26%) [16]. RAS-mutated thyroid cancers were found to be prone to distant metastases in lung and bone, rather than to locoregional lymph node involvement [23]. In our series, mutations in thyroid tissue samples were found in 47% of cases, all with papillary thyroid cancer. Of these mutations, 92% were of BRAF and 8% were of NRAS. These data are in agreement with findings that BRAF is the most common gene mutation (detected in approximately 50% of sporadic papillary thyroid cancers) [24, 25] and NRAS mutations (found with a frequency of 6% by Fukushima et al) [22] are the most prevalently identified additional mutations [21, 22, 24–26]. RET/PTC rearrangement, BRAF, NRAS, and KRAS mutations are considered to be mutually exclusive in papillary thyroid carcinoma [23, 24]. However, Zou et al. [27] reported recently that concomitant mutations of RET/PTC, RAS, or BRAF are a frequent event in advanced papillary thyroid carcinoma and are associated with poor prognosis. The concomitant mutations may represent intratumor heterogeneity and could exert a gene dosage effect to promote disease progression. Of note, we found no case with mutation of KRAS, or with simultaneous mutation of BRAF and NRAS. These findings might also be associated with earlier diagnosis of thyroid cancer in our series. Further studies on the value of preoperative mutation analysis for determining the possibility of malignancy, the occurrence of simultaneous gene mutations in thyroid cancer, and their significance for tumor progression and survival are warranted.

In conclusion, in the federal state of Salzburg, females accounted for 75% of the patients with thyroid cancer and rates peaked at a younger age than in male patients (except for medullary thyroid cancer). The main indication for surgery was a suspicious thyroid nodule. Papillary thyroid cancer was the most frequent tumor type, accounting for 84% of the cases. High frequencies of T1 tumors and cancers with no lymph node involvement were found, especially for papillary thyroid cancer. Male patients had a higher proportion of large tumors and more aggressive forms of thyroid cancer than female patients. Mutations were seen in 47% of the cases and BRAF was the most common mutation. Neither mutations of KRAS nor concomitant mutations of BRAF and NRAS were found.
